# Conventional US, elastography, and contrast enhanced US features of papillary thyroid microcarcinoma predict central compartment lymph node metastases

**DOI:** 10.1038/srep07748

**Published:** 2015-01-13

**Authors:** Yu-Rong Hong, Cao-Xin Yan, Guo-Qaing Mo, Zhi-Yan Luo, Ying Zhang, Yong Wang, Pin-Tong Huang

**Affiliations:** 1 Department of Ultrasound, Second Affiliated Hospital Zhejiang University College of Medicine; 2 Department of Surgery, Second Affiliated Hospital Zhejiang University College of Medicine

## Abstract

Lymph node metastases at the time of diagnosis have a major impact on both therapeutic strategy and tumor recurrence for patients with papillary thyroid microcarcinoma (PTMC). Our objective was to evaluate the usefulness of PTMC characteristics on ultrasonography for predicting central compartment lymph node metastases (CCLNM) of PTMC. One hundred twenty seven patients who underwent surgery for PTMC were enrolled in this study. The relationship between the CCLNM and the characteristics on conventional US, elastographic, and contrast enhanced ultrasound (CEUS) were investigated. Univariate analysis indicated that PTMCs with CCLNM were more often nodule irregular shape, microcalcifications, hyperenhancing or isoenhancing parametric maps, and peak index ≥1 at preoperative US and CEUS than those without CCLNM (*P*< 0.01, 0.05, 0.01 and 0.05 respectively). Multivariate analysis showed that microcalcification (OR:2.378, 95% CI: 1.096–5.158) and hyperenhancement or isoenhancement (OR:2.8, 95% CI: 1.287–6.094) were predictive for the presence of CCLNM. Elastography score was not significantly different between the groups. Our study indicated that preoperative thyroid nodule characteristics on conventional US and CEUS may serve as a useful tool to predict central compartment lymph node metastases in PTMC.

Papillary thyroid microcarcinoma (PTMC) is defined by the World Health Organization (WHO) as a papillary thyroid carcinoma (PTC) 1.0 cm or smaller in its maximal diameter. PTMC has recently been shown to be the most common thyroid malignancy in patients older than 45 years^1^. In most patients, PTMC typically has an indolent course, with a favorable long-term prognosis, and a life expectancy not significantly different from that of the normal population. Nonetheless, some cases of PTMC have aggressive behavior, such as neck lymph nodal metastases, extrathyroidal invasion, local-regional recurrences and distant metastasis^2^,^3^,^4^.

Lymph node metastases at the time of diagnosis have a very significant impact on both type of surgery and tumor recurrence^5^,^6^,^7^. If there are palpable, biopsy-proven, or grossly apparent metastases at the time of surgery, central or lateral lymphadenectomy should be performed. Lymph node metastases are present in about 40% to 60% of patients with PTMC, and the majority of them occur in the central compartment^8^,^9^. Preoperative ultrasound (US), computed tomography (CT) and magnetic resonance imaging (MRI) have relatively low sensitivity (20% to 40%) for detection of lymph node metastases. Particularly, in many patients, lymph node metastases in the central compartment may not show any abnormal finding in preoperative imaging examinations, including on US^10^,^11^.

Some published studies evaluate the biological behavior associated with US features of PTC or PTMC^12^,^13^,^14^. Elastography has been used to evaluate the tissue texture more objectively. By assessing hardness as indicator of malignancy, elastography has recently become an additional tool for thyroid nodule differentiation, in combination with conventional US and fine-needle aspiration cytology (FNAC)^15^,^16^. CEUS has been introduced to investigate tissue microvessel perfusion more sensitively, and it was reported to improve the identification of malignant focal liver lesions^17^,^18^. Previous studies have also demonstrated the feasibility of CEUS for the differentiation of benign and malignant thyroid nodules^19^,^20^. However, few reports have mentioned elastography and contrast-enhanced ultrasound imaging (CEUS) factors to assess the lymph node metastases from PTMC^21^.

In this study, we investigated the relationship between the presence of central compartment lymph node metastases (CCLNM) in patients with PTMC and the conventional US, elastographic, and CEUS characteristics.

## Methods

### Patients

Informed consent was obtained from all patients before their examination, and the local ethics committee and institutional review board approved this prospective study. The methods in this study were performed in accordance with approved guidelines. Follow-up was not conducted in this study. From July 2012 to May 2014, 127 patients (90 women and 37 men) (mean age 43 years; range 18 ~ 77 years) with unilateral PTMC scheduled for surgery were examined using conventional US, elastography and CEUS preoperatively. Ninety-seven patients underwent total thyroidectomy and 30 patients underwent hemithyroidectomy. All patients in this study underwent central node dissection, and 14 of them also underwent unilateral modified radical neck dissection. Lateral neck dissection is indicated for patients in whom lateral neck nodal disease is evident clinically, on preoperative US and nodal FNA or Tg measurement, or at the time of surgery^22^. Seventy-six patients underwent US-guided FNAB preoperatively for several reasons including suspicious US findings (n = 70), a request from the patient or physician (n = 5), lateral neck node metastases from PTC (n = 1). FNAB outcome was: 67 nodules were PTC, 7 nodules were suspicious of PTC, 1 was of undetermined cytology and 1 was negative for malignancy. Fifty-one patients without FNAB preoperatively underwent surgery for suspicious US findings. We performed conventional US, elastography, and CEUS for all patients preoperatively.

### Conventional gray-scale and power-Doppler ultrasound

We used a MyLab90 X-vision (Esaote, Italy) machine equipped with L523 (4 ~ 13 MHz) linear-array transducer for conventional US and elastography, and L522 (3 ~ 9 MHz) linear-array transducer for CEUS. All examinations were performed by the same investigator with fifteen years of experience in thyroid ultrasound.

All selected thyroid nodules were assessed by conventional gray-scale and power-Doppler ultrasound. The echogenicity of the nodule was compared with that of the surrounding parenchyma and was classified as hypoechogenic, isoechogenic, or hyperechogenic. Marked hypoechogenicity was defined by a lower echogenicity than the cervical strap muscle. Shape was classified into three categories: ovoid to round, irregular, and taller than wide. The margin of the nodule was described as well-defined or ill-defined. Calcification within the nodule was classified into four categories: no calcification, microcalcification large and dense calcification, and rim calcification. Microcalcification was defined as hyperechoic spots less than 1 mm in diameter with or without posterior acoustic shadowing. The component of the nodule was classified as solid, predominantly solid (solid portion>50%), predominantly cystic (cystic portion>50%), and cystic. The presence and the pattern of blood flow evaluated by power-Doppler imaging were classified as follows: no power- Doppler signals in the periphery or within the nodule; peripheral vascularity defined as flow in the peripheral position and absent or slight flow in the central part of the nodule; marked intranodular vascularity defined as more flow in the central part of the nodule than at the periphery; mixed vascularity defined as equivalent flow both in the peripheral and central part of the nodule.

### Elastosonography

**Figure 1:**
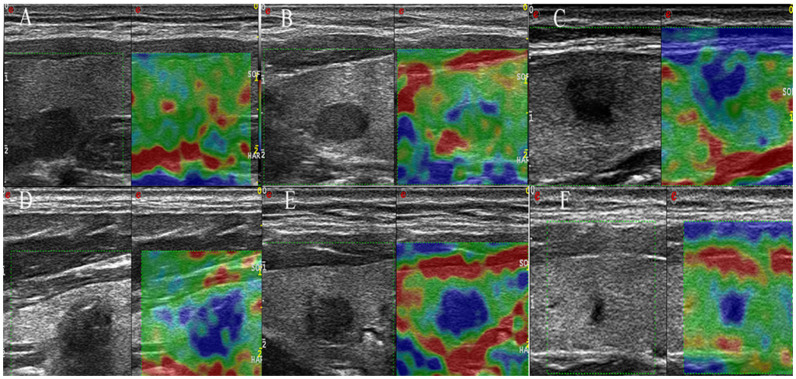
Examples of elastography scoring system for imaging interpretation. Score 1: The entire nodule is evently shaded green (A); Score 2: The nodule is almost completely green, but with some blue spot (B); Score 3: The central part of the nodule is blue, the peripheral part is green (C); Score 4: The nodule is almost completely blue, but with some green spot (D); Score 5: The entire nodule is evently shaded blue (E); Score 6: Both the nodule and the surrounding area are blue (F).

Elastosonography was performed after the conventional ultrasonographic examination by the same investigator. With the scanner switched into the elastographic mode, the probe was placed on the neck with light pressure, and an elastographic region of interest (ROI) was positioned by the operator that included the nodule and sufficient surrounding thyroid tissue to be evaluated. To keep the strain distribution uniform, the probe was pressed to the area with a frequency of 2 to 3 times per second during the cycle of compressing- decompressing in elastography. The real-time elastogram was displayed as an overlay over the gray-scale imaging in a color-coded map: highly elastic tissues (soft) appear in red, less elastic tissues (hard) appear in blue, and intermediate degrees of elastic tissues are shown in green. Elastography images were classified according to the scores by Hong et al.^23^ into a score of 1–6 (Figure 1). In this study, a malignant lesion showing Hong scores of 4 -6 was considered as a “hard” malignancy and remaining scores as “soft”.

### CEUS

CEUS was performed by using Contrast Tuned Imaging technology (CnTI). The acoustic pressure was set at 60 kPa in each patient, and the mechanical index (MI 0.05–0.07) was selected automatically by the system in relation to beam-focus depth. Sulphurhexafluoride (SonoVue®, Bracco, Milan, Italy), was used as ultrasound contrast agent. SonoVue® was injected as an intravenous bolus of 1.2 mL via a 20-gauge cannula into an antecubital vein, followed by a 5 ml saline flush. The thyroid gland including the nodule was scanned for 50 seconds. The video clip was digitally recorded.

### CEUS analysis

**Figure 2:**
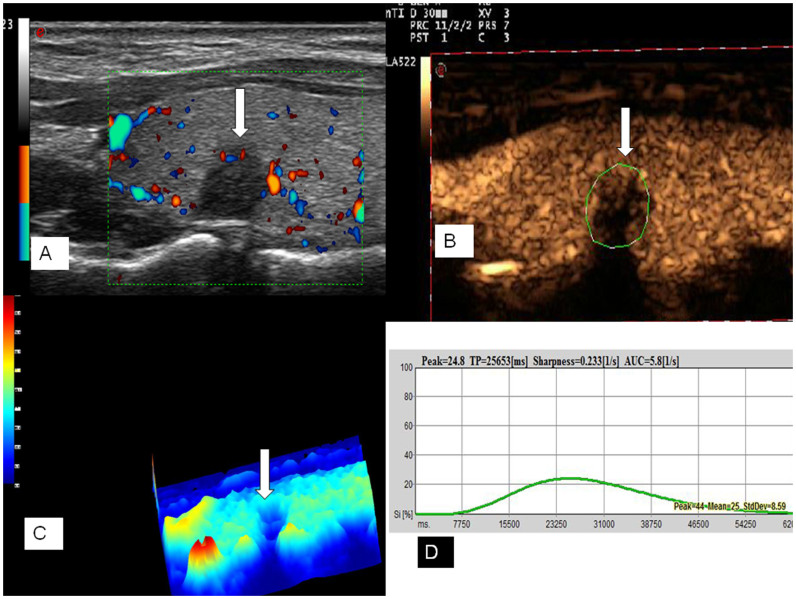
Ultrasound images of a 35-year-old female PTMC patient without CCLNM of the right thyroid lobe. Doppler image showed poor signals in the nodule (A). The CEUS image showed a poor and heterogeneous enhancement (arrow) in the nodule (B). Parametric map showed that the nodule was in blue (arrow), which indicates hypoenhancement with respect to peripheral thyroid parenchyma (C). Numeric values of peak, TP, sharpness, and AUC were automatically calculated based on the time–intensity curve and demonstrated on the top of form (D).

**Figure 3:**
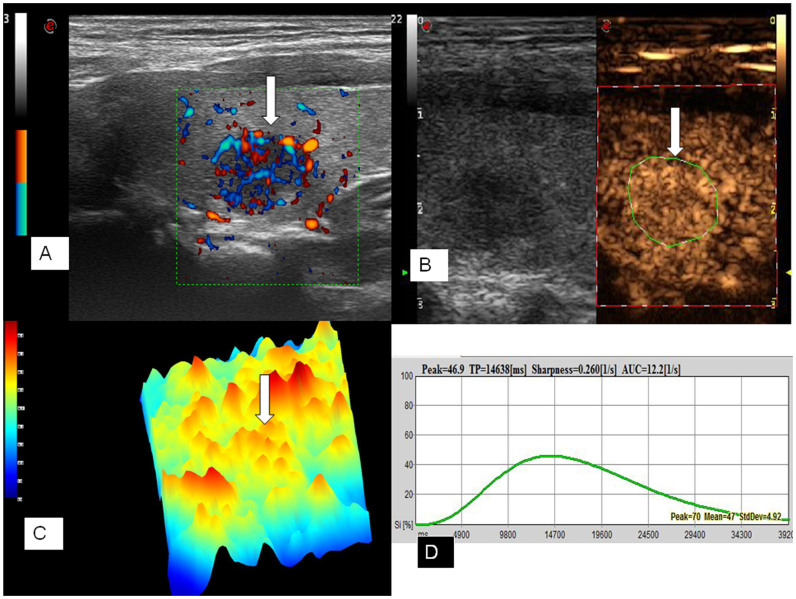
Ultrasound images of a 26-year-old female PTMC patient with CCLNM of the left thyroid lobe. Power Doppler image showed rich blood signals (arrow) in the tumor (A). CEUS image showed diffuse and homogeneous enhancement (arrow) across the whole lesion (B). Parametric map showed that the nodule was in yellow and red (arrow), which indicates hyperenhancement with respect to adjacent thyroid parenchyma (in blue) (C). Numeric values of peak, TP, sharpness, and AUC were automatically calculated based on the time–intensity curve and demonstrated on the top of form (D).

The offline analysis was performed with dedicated software (Qontraxt, EsaoteAmid, Italy). Time-intensity curves within selected ROIs and color maps were acquired. The software enables numeric values to be obtained for each point in the region under examination as the final result; these values are correlated to the quantity of contrast medium that reached the sector in question. A 3D imaging of the perfusion parameters (parametric maps) is obtained of the nodule in a scale of colors varying from red (maximum signal intensity) to blue (minimum signal intensity), passing through the yellow and green. The parametric maps of the thyroid nodules were classified as hyperenhancement, isoenhancement and hypoenhancement (compared with normal thyroid parenchyma color). Hyperenhancement was defined that the color level of nodule was higher than that of parenchyma according to color scale, isoenhancement was defined that the color level of nodule was equal to the parenchyma, hypoenhancement was defined that the color level of nodule was lower than that of parenchyma (Figure 2, 3). In each case, signal intensity of the nodule was measured in decibels (dB). Nodule and parenchyma thyroid tissue values of peak intensity (PI, expressed as a percentage, maximum intensity = 100%), time to peak (TP, expressed in seconds), sharpness of the bolus transit (expressed in seconds^−1^), and area under the curve (AUC, expressed in seconds^−1^) were calculated. Because ultrasound enhancement measurements are subject to physiological effects and individualized ultrasound settings, we chose to include relative nodule enhancement to surrounding thyroid parenchyma enhancement data in our analysis. PI, TP, sharpness and AUC are reported as indexes (Peak index, TP index, sharpness index, AUC index) derived from the ratio between the values from the ROI of the nodule and the ROI of normal thyroid parenchymal tissue.

### Statistical analysis

SPSS version 16.0 (SPSS Inc. Chicago,IL USA) was used for the statistical analysis. Continuous data are presented as mean (standard deviation) and compared by using Student's t test. Categorical data are presented as percentages and compared using the Chi-square test and Fisher's exact test. The Pearson chi-squared test and Fisher's exact test were used to compare the categorical data. The rankings of valuable indicators were assessed according to the odds ratios (ORs). All risk ultrasound features that proved to be statistically significant on univariate analysis were analyzed to assess independent association with CCLNM using multivariate logistic regression.

## Results

### Demographic and clinicopathologic characteristics

Of 127 patients, 39 (30.7%) had CCLNM, and 11 (8.7%) had ipsilateral lymph node metastases on pathology. Metastasis to the ipsilateral cervical compartment without metastasis to the central cervical compartment was found in 5 patients (3.9%).

Patients clinical characteristics are shown in Table 1. For unilateral multifocal microcarcinoma, the analysis was first performed according to the largest tumor. Total tumor diameter (TTD) was then calculated as the sum of the maximal diameter of each lesion on pathological examination and used for further analysis. Univariate analysis indicated that patients with CCLNM showed a significantly higher proportion of patients aged ≤45 years than patients without CCLNM (76.9% vs 47.7%, *P* = 0.002). While other characteristics have no significant difference between two groups (*P*>0.05, for all).

### Ultrasonographic findings of PTMCs with CCLNM

As shown in Table 2, PTMCs with CCLNM were more often nodule irregular shape, microcalcifications, hyperenhancing or isoenhancing parametric maps, and peak index ≥1 at preoperative US and CEUS than those without CCLNM (*P*< 0.01, 0.05, 0.01 and 0.05 respectively). While the proportion of TP index≥1, Sharpness index ≥1, and AUC index ≥1 were not significantly different between the two groups (*P*>0.05, for all). Twenty-one (53.8%; 21 of 39) patients with CCLNM and 26 29.5%; 26 of 88) patients without CCLNM had irregular shape. Twenty (51.2%; 20 of 39) patients with CCLNM and 27(30.7%; 27 of 88) patients without CCLNM had microcalcifications. Twenty-four (61.5%; 24 of 39) patients with CCLNM and 27(36.4%; 32 of 88) patients without CCLNM had hyperenhancing or isoenhancing parametric maps. Seven (17.9%; 7 of 39) patients with CCLNM and 5 (5.7%; 5 of 88) patients without CCLNM had the peak index ≥1. The sensitivity and specificity in the evaluation of CCLNM were 51% and 69%, respectively in cases with microcalcification, 54% and 70%, respectively in cases with irregular shape, 62% and 64%, respectively in cases with hyperenhancing or isoenhancing parametric maps, and 18% and 94%, respectively in cases with peak index≥1.

### Multivariate logistic regression analysis

Multivariate logistic regression showed that microcalcifications (OR = 2.378; 95%CI 1.096–5.158; *P* = 0.043) and hyperenhancing or isoenhancing parametric maps (OR = 2.8; 95%CI 1.287–6.094; *P* = 0.026) were predictive for the presence of CCLNM (Table 3).

## Discussion

High-resolution ultrasound is regarded as a first-line method for the preoperative diagnosis of PTC. Recent studies showed that US features may be also useful in predicting the biological behavior of PTC.

Nam et al.^12^ retrospectively evaluated the ultrasonographic characteristics of 354 PTCs. Malignant-looking PTCs (M-PTCs) were defined as those showing at least 1 accepted ultrasonographic criterion: taller than wide shape, marked hypoechogenicity, microcalcifications, and infiltrative borders. Benign-looking PTCs (B-PTCs) were defined as tumors without any of these criteria. They reported that M-PTCs have more aggressive biological behavior than B-PTCs, including extrathyroidal extension, LN metastases, and advanced stage. Kim et al.^13^ evaluated 354 patients who underwent surgery for PTC (≤2 cm). They found that tumor size, shape, margin, echogenicity, and contact with the thyroid capsule of ultrasonographic characteristics were significantly different between the patients with and without CCLNM. Multivariate logistic regression showed that a tumor diameter greater than 1 cm (compared to a tumor diameter 1 cm or less) and hypoechogenicity predicted the presence of CCLNM. Furthermore, the diagnostic accuracy of the US criteria depends on tumor size^23^,^24^,^25^. Our previous study^23^ showed that microcalcifications were more frequent in large (>1 cm) than in small malignant nodules (≤1 cm).

For malignant nodules, the frequency of irregular shape had no significant difference between the nodules >1 cm and nodules ≤1 cm. The echogenicity does not depend on nodule size^23^,^24^,^25^. In this study, on univariate analysis we found that irregular shape and microcalcification were more frequent in PTMC with CCLNM than without CCLNM, but we did not find a correlation between echogenicity and CCLNM. Multivariate logistic regression showed that microcalcification was predictive for the presence of CCLNM. In histopathology, microcalcification is thought to represent psammoma bodies, typically found in PTC. They may be associated with the aggressive behaviors of PTMCs.

Elastography, a noninvasive technique, has been introduced to evaluate the texture of the tissue and is useful in differentiating benign and malignant thyroid nodules^26^,^27^,^28^,^29^. Moon et al.^21^ also reported that a hard malignancy (Rago scores of 4–5) was an independent factor for predicting pathologic extrathyroidal extension on pathology, but was not a factor for predicting central and lateral lymph node metastasis on pathology of patients with PTMC. In this study, we also find that the stiffness of a malignancy was not associated with CCLNM.

CEUS reveals the microvascularity of tumor tissue, obtaining a spatial and temporal resolution superior to the traditional color and power Doppler techniques. Moreover, time-intensity curves can be calculated, allowing both qualitative and quantitative evaluation^30^,^31^,^32^,^33^. There are few reports on the association of the CEUS characteristics with the biologic behavior of PTC. In our study, 37% of patients with PTMC showed hyperenhancing or isoenhancing parametric maps, 63% showed hypoenhancing parametric maps. Hyperenhancing or isoenhancing parametric maps suggested that the tumor tissue may have equal or more blood supply than the surrounding thyroid parenchyma, and hypoenhancing parametric maps suggested that the tumor tissue may have less blood supply than the peripheral thyroid parenchyma. Peak index ≥1 means that the peak intensity of the tumor was higher than the peripheral thyroid parenchyma. In this study hyperenhancing or isoenhancing parametric maps, and peak index ≥ 1 of the preoperative CEUS findings were more frequent in patients of PTMC with CCLNM than without CCLNM on univariate analysis. Multivariate logistic regression showed that microcalcification was predictive for the presence of CCLNM. Thus, the blood supply of the tumor may be associated with the biological behavior of PTMC.

Numerous previous studies have mentioned various factors that influence the aggressive biologic behaviors of PTC^34^,^35^,^36^,^37^,^38^. Zhao et al.^34^ reported that the frequency of lymph node metastases was significantly higher in multifocal PTMCs with TTD>1 cm than unifocal tumors with diameter ≤1 cm. Multifocal PTMC with TTD>1 cm has a similar risk of lymph node metastases as a papillary cancer. However, in this study, the difference of TTD was not significantly between the patients with PTMC with and without CCLNM (*P* = 0.107). Dvorkin et al.^35^ demonstrated that Hashimoto thyroiditis was associated with a less aggressive form of thyroid cancer and a better long-term outcome. However, 10.3% of PTMC with CCLNM and 18.2% of PTMC without CCLNM suffered from Hashimoto thyroiditis in this study, and this difference was not significant (*P* = 0.258).

Our study did have several limitations. First, most of the patients in this study had suspicious US findings for malignancy, and there may have been selection bias. Second, we only evaluated the association of CCLNM and tumor US findings. The aggressive biological behavior of PTMC includes lateral lymph node metastases, extrathyroidal invasion, distant metastases, and recurrence. Although the majority of lymph node metastases are within the central neck, prediction of lateral cervical neck metastases may have important clinical significance. If these lateral cervical neck metastases are detected preoperatively, a lateral neck dissection may improve outcome. Third, this study did not include long-term follow-up. A large series study with a prospective design and long-term follow-up is needed.

In conclusion, nodule shape, microcalcification, hyperenhancing or isoenhancing parametric maps, and peak index in the preoperative US and CEUS findings were significantly associated with CCLNM of patients with PTMC. Thus, US and CEUS features at the time of diagnosis can serve as a useful tool to predict the biological behavior of PTMC.

## Author Contributions

P.H. designed this study. Y.H., G.M., Z.L. and Y.W. acquired the data. P.H., Y.H., C.Y. and Y.Z. interpreted the data. P.H. and Y.H. wrote the main manuscript text. All authors reviewed the manuscript.

## Figures and Tables

**Table 1:** Univariate Analysis of Clinical characteristics of Patients with or without CCLNM

Characteristics	CCLNM		*P* Value
	Yes(*n* = 39)	No(*n* = 88)	
Male sex	12	25	0.787
TTD, cm	0.78 ± 0.48	0.65 ± 0.35	0.107
Age≤45 y	30 (76.9)	42(47.8)	0.002
Tumor size ≥ 0.5 cm	33	65	0.183
Multifocality	4	6	0.507
Extrathyroidal extension	6	14	0.94
Hashimoto thyroiditis	4	16	0.258
Distant metastasis	0	0	

CCLNM, central compartment lymph node metastases.

TTD: total tumor diameter.

**Table 2:** Ultrasonographic nodule characteristics based on CCLNM

Parameters	Characteristics	CCLNM		*P* Value
		Yes(*n* = 39)	No(*n* = 88)	
Size,cm		0.79 ± 0.25	0.70 ± 0.24	0.073
Shape	ovoid to round	12	40	
	irregular	21(53.8)	26(29.5)	0.009
	taller than wide	6	22	0.201
Margin	well-defined	2	11	
	ill-defined	37	77	0.206
Echogenicity	markedly hypoechoic	8	19	0.891
	hypoechoic	28	61	0.796
	Isoechoic	2	6	
	hyperechoic	1	2	
Calcification	absent	17	58	
	microcalcification	20(51.2)	27(30.7)	0.027
	macrocalcifications	2	3	
	rim calcification	0	0	
Vascularity	no vascularity	2	14	0.091
	peripheral vascularity	25	48	
	marked intranodular vascularity	2	3	
	mixed vascularity	10	23	
Elastosonography	hard	33	74	0.94
	soft	6	14	
Perfusion pattern	centripetal	23	59	0.38
	centrifugal	16	29	
Enhancement type	hyper-or isoenhancment	24(61.5)	32(36.4)	0.008
	hypoenhancement	15	56	
Peak index	≥1	7(17.9)	5(5.7)	0.029
TP index	≥1	31	66	0.583
Sharpness index	≥1	26	60	0.866
AUC index	≥1	9	20	0.965

CCLNM: central compartment lymph node metastases.

TP: time to peak.

AUC: area under the curve.

**Table 3:** Multivariate analysis of association of central compartment lymph node metastasis and US characteristics

Characteristic	OR	95%CI	*P* value
**Irregular shape**	2.782	1.277–6.06	0.237
**Microcalcification**	2.378	1.096–5.158	0.043
**Hyper-or isoenhancement**	2.8	1.287–6.094	0.026
**Peak index**	3.631	1.074–12.275	0.236
